# Retinal pigment epithelial cell necroptosis in response to sodium iodate

**DOI:** 10.1038/cddiscovery.2016.54

**Published:** 2016-07-04

**Authors:** J Hanus, C Anderson, D Sarraf, J Ma, S Wang

**Affiliations:** 1 Department of Cell and Molecular Biology, Tulane University, 2000 Percival Stern Hall, 6400 Freret Street, New Orleans, LA 70118, USA; 2 Department of Ophthalmology, Tulane University, 1430 Tulane Avenue, SL-69, New Orleans, LA 70112, USA

## Abstract

Age-related macular degeneration (AMD) is a degenerative disease of the retina and the leading cause of blindness in the elderly in developed countries. The late stage of dry AMD, or geographic atrophy (GA), is characterized by extensive retinal pigment epithelium (RPE) degeneration. The underlying molecular mechanism for RPE cell death in GA remains unclear. Our previous study has established that RPE cells die predominantly from necroptosis in response to oxidative stress *in vitro*. Here, we extend our study and aim to characterize the nature of RPE cell death in response to sodium iodate (NaIO_3_) *in vitro* and in a NaIO_3_-induced retina degeneration mouse model. We found that NaIO_3_ induces RPE necroptosis *in vitro* by using a combination of molecular hallmarks. By using TUNEL assays, active caspase-3 and HMGB1 immunostaining, we confirmed that photoreceptor cells die mainly from apoptosis and RPE cells die mainly from necroptosis in response to NaIO_3_ *in vivo*. RPE necroptosis in this model is also supported by use of the RIPK1 inhibitor, Necrostatin-1. Furthermore, using novel RIPK3-GFP transgenic mouse lines, we detected RIPK3 aggregation, a hallmark of necroptosis, in the RPE cells *in vivo* after NaIO_3_ injection. Our findings suggest the necessity of re-evaluating RPE cell death mechanism in AMD models and have the potential to influence therapeutic development for dry AMD, especially GA.

Age-related macular degeneration (AMD) is a degenerative disorder of the macula and the leading cause of irreversible central vision loss in the elderly population in the developed countries.^1^ The dry form of AMD is characterized by a yellow deposit called ‘drusen’ under the retina at the early stage and geographic atrophy (GA) at the late stage. GA is manifested in scattered or confluent areas of degeneration of retina pigment epithelial (RPE) cells. RPE degeneration is thought to result in the degeneration of the overlying photoreceptors and eventually vision loss.

Age is the most consistent risk factor associated with AMD. Genetic factors, oxidative stress, inflammation, and ethnicity are considered to be contributors to the pathogenesis of AMD.^2^ Among them, oxidative stress has been suggested as a critical component of AMD pathogenesis.^3^ Cigarette smoking, which induces systemic oxidative stress, has been demonstrated to be a significant risk factor for AMD. Clinical studies have shown that the progression of AMD can be slowed with antioxidant vitamins and zinc supplements.^4,5^ The complete pathological mechanism underlying dry AMD has not been completely understood, and the disease is currently untreatable.

Sodium iodate (NaIO_3_) injection has been extensively used as a pre-clinical model of RPE dystrophy and GA.^6^ NaIO_3_-induced retinal degeneration displays two features similar to AMD. First, low doses lead to a patchy loss of the RPE cells leaving spots void of autofluorescence as in GA. Second, the RPE loss not only affects the photoreceptors but also the underlying choriocapillaris.^7^ NaIO_3_ is thought to directly affect the RPE cells with secondary effects on photoreceptors and the choriocapillaris and has been shown to induce the production of reactive oxygen species contributing to damages in RPE cells.^8,9^ Other effects of NaIO_3_ on RPE cells include: inhibition of enzyme activity (e.g., triose phosphate dehydrogenase, lactate dehydrogenase) in RPE cells, disruption of the blood–retina barrier, and increased conversion of glycine to potentially toxic glyoxylate by melanin.^10–12^

Two major types of cell death, apoptosis and necroptosis, occur in response to oxidative stress.^13^ Apoptosis is characterized by maintenance of the plasma membrane, chromatin condensation and fragmentation, and caspase activation. Necroptosis is a regulated form of necrosis mediated by receptor-interacting protein kinases (RIPK).^14^ In contrast to apoptosis, necroptosis is characterized by ATP depletion, rupture of the plasma membrane, and release of necroptosis-specific cytokine HMGB1 to activate inflammatory response.^15,16^ Owing to the different implications in inflammatory response between apoptosis and necroptosis, to develop targeted therapy for AMD, it is crucial to clarify the mechanism of RPE cell death in response to oxidative stress and in AMD. We recently found that the molecular features of apoptosis were not observed in RPE cells in response to H_2_O_2_ or tBHP treatment.^17^ Instead, cardinal features of necroptosis, including ATP depletion, RIPK3 aggregation, and the release of HMGB1 from the nucleus were detected. Inhibition of RIPK activity by necrostatins or downregulation of RIPK3 by siRNAs largely rescued oxidative stress-induced RPE death. Our results suggest that RPE necroptosis is the predominant mechanism of RPE cell death in response to oxidative stress *in vitro*.

Here, we extend our previous study and characterize the nature of RPE cell death in both *in vitro* and *in vivo* NaIO_3_ models. We provide evidence that NaIO_3_ induces RPE necroptosis, but not apoptosis *in vitro*. We also utilize a NaIO_3_-induced RPE degeneration model to analyze the molecular changes associated with RPE and photoreceptor cell death *in vivo*, and confirm photoreceptor apoptosis and RPE necroptosis in response to NaIO_3_ *in vivo*. RPE necroptosis in the model is supported by use of RIPK1 inhibitor, Necrostatin-1. By using novel RIPK3-GFP transgenic mouse lines, we detected RIPK3 aggregation, a hallmark of necroptosis, in the RPE cells after retro-orbital NaIO_3_ injection, further confirming RPE necroptosis *in vivo*.

## Results

### Sodium iodate-induced RPE necroptosis *in vitro*

The NaIO_3_ animal model has been widely used as a pre-clinical model of RPE dystrophy and atrophic AMD. Surprisingly, very few studies documented the effect of NaIO_3_ in RPE cells *in vitro*.^18,19^ We first determined the half maximal effective concentration (EC50) of NaIO_3_ in confluent ARPE-19 cells. After testing the RPE cell survival at 24 h after different concentrations of NaIO_3_ (0–75 mM) treatment, the EC50 of NaIO_3_ in ARPE-19 cells was calculated to be 10.5 mM (Supplementary Figure 1); 10 mM NaIO_3_ was then selected for the subsequent *in vitro* experiments.

**Figure 1:**
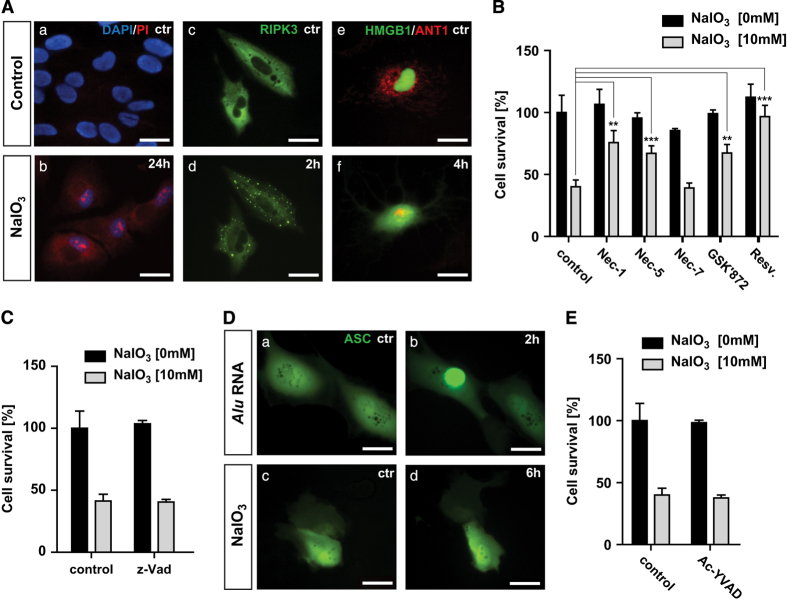
Evidence for sodium iodate-induced RPE necroptosis *in vitro*. (**A**) NaIO_3_ (10 mM) treatment induces changes to cell membrane permeability in non-fixed ARPE-19 cells shown by staining with PI (a and b). Activation of RIPK3 after 2 h of 10 mM NaIO_3_ treatment in RIPK3-GFP-transfected ARPE-19 cells (c and d). Mitochondrial network was tracked by ANT1-RFP and passive release of HMGB1 from the nucleus by HMGB1-YFP at 4 h after NaIO_3_ treatment (e and f). (**B**) ARPE-19 cells were treated with 200 *μ*M Nec-1, -5, -7; 3 *μ*M GSK’872, or 25 *μ*M resveratrol for 24 h before 10 mM NaIO_3_ treatment. Cell viability was measured by MTT assay at 24 h. (**C**) ARPE-19 cells were treated with 50 *μ*M z-VAD for 24 h before 10 mM NaIO_3_ treatment. Cell viability was measured by MTT assay at 24 h later. (**D**) Activation of inflammasome was tracked by transfection of ASC-GFP-expressing plasmid in ARPE-19 cells. Alu RNA (5 ng) was used as a positive control for inflammasome activation (a and b). Treatment with 10 mM NaIO_3_ did not induce inflammasome activation (c and d). (**E**) ARPE-19 cells were treated with 50 *μ*M Ac-YVAD for 24 h before 10 mM NaIO_3_ treatment. Cell viability was measured by MTT assay at 24 h later. *P<0.05; **P<0.01; ***P<0.001. The scale bar is 25 *μ*M.

To examine the nature of NaIO_3_-induced RPE cell death *in vitro*, we tested cell membrane permeability to propidium iodide (PI), an established hallmark of necrosis. Confluent ARPE-19 cells were subjected to DAPI/PI staining at 24 h after treatment with 10 mM of NaIO_3_. Strong cytoplasmic and nuclear staining with PI was observed in the treated but not the control cells (Figure 1A (a and b)). We have previously shown that necrotic ARPE-19 cells show RIPK3 aggregation and HMGB1 nuclear release.^17^ We analyzed the cellular distribution of RIPK3 by transfecting ARPE-19 cells with an RIPK3-GFP-expressing plasmid. RIPK3 was evenly distributed in the cytoplasm under normal condition, but formed punctuates in the periphery region of cells within 2 h of 10 mM NaIO_3_ treatment, indicating necrosome formation (Figure 1A (c and d)). HMGB1 distribution and mitochondria morphology were visualized in ARPE-19 cells after co-transfecting with plasmids expressing HMGB1-YFP and ANT1-RFP. HMGB1 is a chromatin structural protein detected inside the nucleus. Within 4 h of NaIO_3_ treatment, HMGB1 was released to the cytoplasm as indicated by the distribution of HMGB1-YFP signal (Figure 1A (e and f)). The mitochondria form a tubular network as visualized after ANT1-RFP transfection. At 4 h of NaIO_3_ treatment, mitochondrial network was fragmented and clustered in the perinuclear region (Figure 1A (e and f)). Taken together, these results suggest that necroptosis but not apoptosis mainly accounts for cell death in ARPE-19 cells in response to NaIO_3_ treatment.

To further confirm the necrotic nature of cell death in RPE cells in response to NaIO_3_ treatment, ARPE-19 cells were treated with different cell death inhibitors before exposure to NaIO_3_. We pretreated APRE-19 cells with 200 *μ*M of Nec-1, -5, or -7 for 24 h and exposed them to 10 mM of NaIO_3_. As a positive control, resveratrol (25 *μ*M) rescued up to 93% of ARPE-19 cells from NaIO_3_-induced death.^19^ Nec-1, a direct RIPK1 inhibitor, increased ARPE-19 cell survival from 47 to 75%. Nec-5, an indirect RIPK1 inhibitor, increased ARPE-19 cell survival to 67%, while Nec-7 that targets RIPK1-independent necrosis pathways had no effect on ARPE-19 viability. In addition, GSK’872, a specific RIPK3 inhibitor, protected up to 67% ARPE-19 cells from NaIO_3_-induced cell death (Figure 1B). However, z-VAD, a pan-caspase inhibitor, failed to protect ARPE-19 cells from NaIO_3_-induced death (Figure 1C). These observations indicate that NaIO_3_-induced RPE cell death is mainly necroptosis that depends on RIPK1 and RIPK3.

RIPK3 activation has been recently shown to be implicated in the induction of pyroptosis, a cell death pathway that relies on caspase-1 activation and inflammasome formation.^20^ To test whether NaIO_3_ can induce inflammasome activation in RPE cells, ARPE-19 cells were transfected with ASC-GFP, a component of the inflammasome.^21^ ASC-GFP-transfected ARPE-19 cells showed uniform cytoplasmic ASC-GFP expression. As a positive control, Alu RNA treatment induced inflammasome formation in ARPE-19 cells as shown by aggregated cytoplasmic ASC-GFP foci (Figure 1D (a and b)).^22^ When ARPE-19 cells were treated with NaIO_3_ for 0–24 h, aggregated ASC-GFP foci were not observed, indicating no inflammasome formation (Figure 1D (c and d)). To further confirm this observation, ARPE-19 cells were treated with 50 *μ*M caspase-1 inhibitor Ac-YVAD for 24 h prior to NaIO_3_ treatment. No rescue of ARPE-19 cells was detected arguing against the involvement of pyroptosis in RPE cell death in response to NaIO_3_ (Figure 1E).

Taken together, our observations indicate that NaIO_3_ induces mainly RIPK1- and RIPK3-dependent necroptosis in ARPE-19 cells *in vitro*.

### Morphological changes in the mouse retina after retro-orbital NaIO_3_ administration

**Figure 2:**
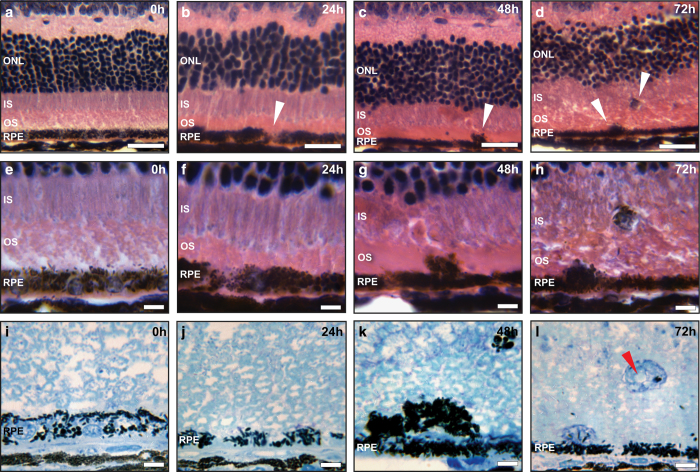
Histological staining of the mice retina after NaIO_3_ administration. (**a**–**d**) Hematoxylin and eosin staining of paraffin-embedded retinal cross sections of 20 mg/kg NaIO_3_-treated C57BL/6J mice, at different time points. At 24 h post administration, loss of RPE pigmentation was visible (arrowheads); RPE swelling and breaking of RPE monolayer was observed at 48 and 72 h, respectively (arrowheads). Scale bar is 25 *μ*m. (**e**–**h**) Areas indicated by arrowheads at higher magnification. Scale bar is 10 *μ*m. (**i**–**l**) Semi-thin sections and methylene blue staining revealed more details of the degenerated but still partially pigmented RPE cells with visible cell vacuolization at 72 h (arrowhead). Scale bar is 10 *μ*m. ONL: photoreceptor outer nuclear layer, IS: inner segments, OS: outer segments. Results shown here are representative from six C57BL/6J mice at each time point.

Although NaIO_3_ administration has been widely used to induce RPE degeneration, there has been little in-depth analysis regarding the nature of RPE cell death *in vivo* in this model. NaIO_3_ has been shown to induce reactive oxygen species in RPE cells, making it an excellent model to study oxidative stress *in vivo*.^23^ We adopted a retro-orbital injection of low dose NaIO_3_ (20 mg/kg) to mouse retina to examine the nature of RPE cell death *in vivo*, with a focus to capture the early events leading to RPE death.^24–26^ H&E staining and methylene blue staining were used to validate the effect of NaIO_3_ administration in RPE cells. In control animals treated with saline, the RPE cell layer was evenly pigmented (Figure 2a and e) with RPE polarization noticeable in semi-thin plastic sections (Figure 2i). Furthermore, photoreceptor outer nuclear layer, inner segments, and outer segments were well organized. At 24 h after 20 mg/kg NaIO_3_ administration, RPE cells showed signs of loss of pigmentation with no visible effect on photoreceptor morphology (Figure 2b *arrowhead*, Figures 2f and j). At 48 h post injection, patchy RPE degeneration was observed: RPE appeared swollen, arrangement of photoreceptor segments was disorganized, and inner segments lost their regular organization (Figure 2c *arrowhead*, Figures 2g and k). At 72 h post injection, RPE cells appeared swollen, vacuolized, and started to break off from the RPE layer. Disorganization of outer nuclear layer, inner segments, and outer segment continued to progress reflecting photoreceptors cell death (Figure 2d *arrowhead*, Figures 2h and l). Throughout the time points, the RPE cell layer became progressively thinner. Taken together, our morphological analyses suggest necrotic death of RPE cells in the low-dose NaIO_3_ model, and validate this system for further molecular studies.

### Molecular characteristics of retinal cell death upon retro-orbital NaIO_3_ administration

**Figure 3:**
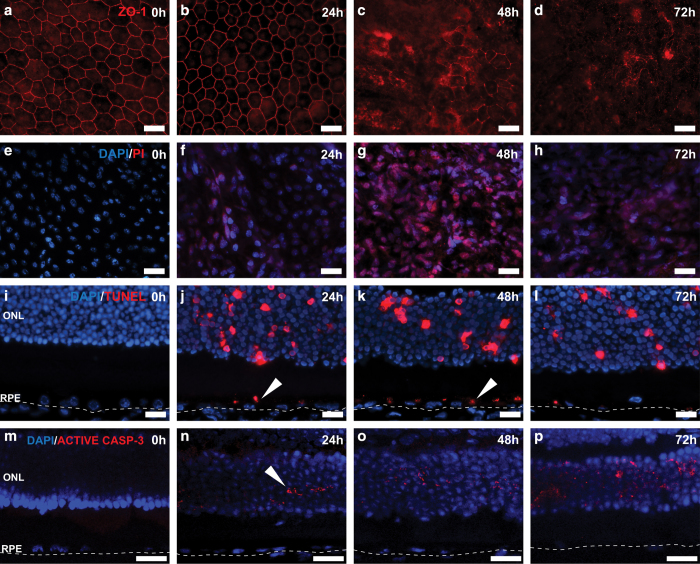
Molecular hallmarks of RPE degeneration in C57BL/6J mice RPE after retro-orbital NaIO_3_ administration. (**a**–**d**) Tight junctions in the central retina were visualized by ZO-1 staining. At 48 h post 20 mg/kg NaIO_3_ administration, compromise of tight junctions was visible indicating impairment of the blood–retina barrier (*n*=6 each). (**e**–**h**) Flatmount DAPI and PI staining highlighted necrotic RPE cells as early as 24 h post NaIO_3_ administration. At 72 h, areas with diffused PI signal were seen (*n*=6 each). (**i**–**l**) TUNEL staining was used to highlight dying cells in the retina. Both RPE cells and photoreceptors were TUNEL-positive as early as 24 h post 20 mg/kg NaIO_3_ administration (*n*=6 each). (**m**–**p**) Active caspase-3 staining was observed only in the photoreceptor layer at all analyzed time points (*n*=5 each). The scale bar is 25 *μ*M.

To dissect the molecular changes in the retina after retro-orbital NaIO_3_ administration, tight junctions were visualized by immunostaining with antibody to ZO-1, a tight junction-associated protein. NaIO_3_ administration resulted in the loss of RPE monolayer properties as visualized by ZO-1 staining, at 48 h after NaIO_3_ injection, indicating a compromise of the blood–retina barrier (Figure 3a–d).

Retro-orbital PI injection was further used to confirm RPE necrosis in this model. Retinal flatmount revealed the appearance of PI-positive RPE cells as early as 24 h after NaIO_3_ injection (Figures 3e–h). At 48 h post injection, most RPE cells were PI-positive. The PI staining was observed in both the cytoplasm and nucleus of the RPE cells. A similar effect was observed at 72 h post injection. This DAPI/PI staining suggests RPE necrosis within 72 h of retro-orbital NaIO_3_ injection.

Next, we used a combination of TUNEL assay and active caspase-3 staining to examine the nature of RPE cell death in this model. TUNEL-positive photoreceptor cells (Figures 3i–l) were abundantly detected as early as 24 h post NaIO_3_ injection, and persisted at 72 h. TUNEL-positive RPE cells were detected at 24 h, and appeared to be more abundant at 48 h. TUNEL-positive RPE cells decreased at 72 h (Figures 3i–l, *arrowheads*). However, active caspase-3 staining was observed only in photoreceptor layer but not in RPE cells (Figures 3m–p, *arrowheads*).

Taken together, RPE cells in the retro-orbital NaIO_3_ model showed loss of ZO-1 staining, positive PI and TUNEL staining, and negative active caspase-3 staining. These features indicate that RPE cells die from necrosis but not apoptosis *in vivo* in response to oxidative injury induced by low dose NaIO_3_.

### Sodium iodate induces RIPK3 aggregation and RPE necroptosis

**Figure 4:**
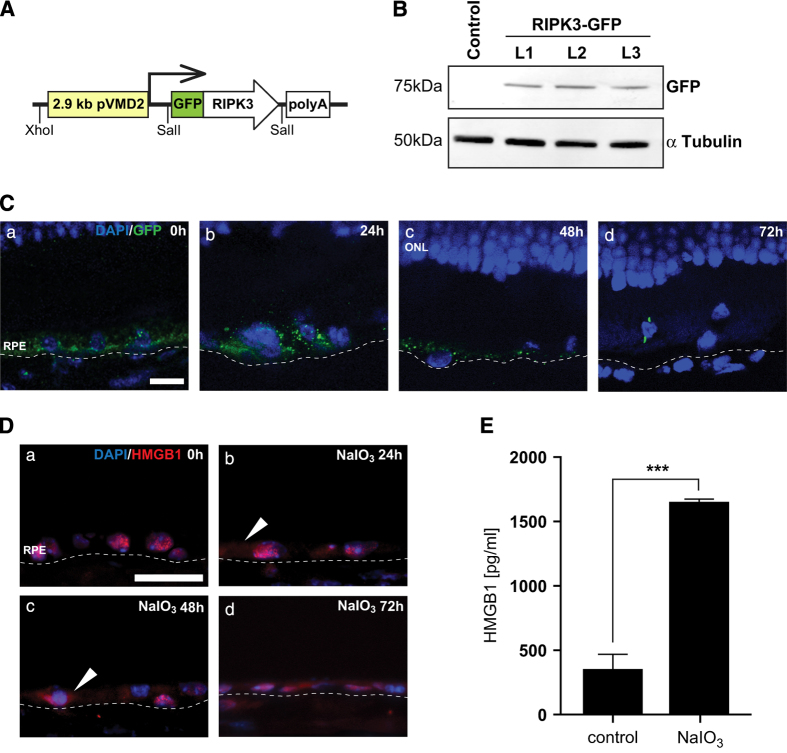
Characterization of the necroptosis *in vivo* by using pVMD-RIPK3-GFP transgenic mice and by visualizing HMGB1 release. (**A**) Schematics of the construct for transgenic mice. (**B**) Confirmation of transgene expression in three lines (L1-L3) by western blot. (**C**) RIPK3-GFP expression in the RPE layer of RIPK3-Tg mice was visualized by GFP staining (a), RIPK3-GFP aggregation as a result of 20 mg/kg NaIO_3_ was visible at 24 and 48 h (b and c) post administration. Very few GFP staining was observed at 72 h (d) (*n*=5 each). The scale bar is 10 *μ*M. (**D**) Release of HMGB1 from the nucleus of RPE cells was visualized by HMGB1 staining. In normal RPE cells, HMGB1 is located in the nucleus (a), and 24 h after 20 mg/kg NaIO_3_ administration, HMGB1 was released into the cytoplasm (b, arrowheads). At 48 and 72 h, extranuclear HMGB1 can be observed in the RPE cell layer (c and d, arrowheads) (*n*=5 each). The scale bar is 25 *μ*M. (**E**) HMGB1 release was measured in the vitreous humor using ELISA (*n*=3 each). ***P<0.001.

RIPK3 aggregation and the formation of the necrosome is a critical step in necroptosis. Although we and others have successfully established RIPK3 aggregation as a necrotic hallmark *in vitro*, detection of RIPK3 aggregation *in vivo* has been challenging. To further confirm whether RPE cells undergo necroptosis *in vivo*, we have made transgenic mouse lines expressing human RIPK3 fused to a GFP protein under the control of the RPE-specific VMD2 promoter (Figure 4A). The expression of RIPK3-GFP in the mouse lines was verified by western blot (Figure 4B).

Immunostaining using anti-GFP antibody confirmed the expression of RIPK3-GFP specifically in the RPE cell layer of the transgenic mice (Figure 4C (a)). RIPK3 aggregation in the cytoplasm as shown by GFP staining was observed at 24 and 48 h after retro-orbital injection (Figure 4C (b and c)). RIPK3 aggregation was not observed at 72 h post injection, consistent with the notion that RIPK3 aggregation is an early event in necroptosis (Figure 4C (d)).

Necroptosis is also associated with the release of HMGB1, a member of the DAMP molecules, to the cytoplasm and outside of the cells. In the normal RPE cells, HMGB1 is localized in the nucleus (Figure 4D (a)). At 24 h post retro-orbital NaIO_3_ administration, extranuclear HMGB1 was detected in the RPE cell layer by immunostaining (Figure 4D (b-d)). Consistently, the level of HMGB1 was also significantly increased in the vitreous humor of the treated mice compared with the control by ELISA analyses (Figure 4E).

These experiments established that NaIO_3_ induces RIPK3 aggregation and HMGB1 release in mouse RPE cells *in vivo*, providing strong evidence of RPE necroptosis in this model.

### Necrostatin-1 protects RPE from degeneration induced by NaIO_3_*in vivo*

**Figure 5:**
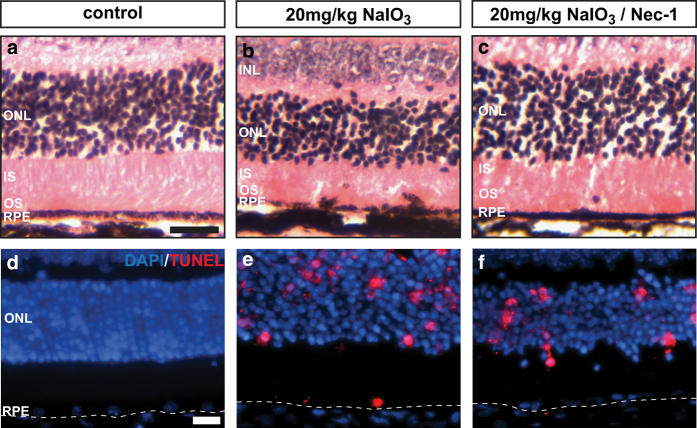
Inhibitor of necroptosis prevents RPE degeneration. (**a**–**c**) Hematoxylin and eosin staining of paraffin-embedded retinal cross sections at 48 h post 20 mg/kg NaIO_3_ or NaIO_3_/Nec-1 retro-orbital administration. (**d**–**f**) TUNEL analysis of RPE/photoreceptor cell death. The scale bar is 25 *μ*M. *n*=6 each.

Our *in vitro* and *in vivo* data have established that RPE cells die from necroptosis after exposure to NaIO_3_. To further confirm RPE necroptosis in response to oxidative stress, we tested whether Nec-1, a potent and selective RIPK1 inhibitor, can inhibit RPE cell death in this model. Nec-1 (400 *μ*M) was injected by retro-orbital injection at the time of NaIO_3_ administration. Mouse eyes were harvested 48 h post Nec-1/NaIO_3_ administration. Morphological analysis revealed that Nec-1 protected RPE cells from degeneration and partially maintained organization of the inner segments of the photoreceptors (Figures 5a–c). Nec-1 significantly decreased TUNEL positivity in RPE cells, while photoreceptors retained TUNEL positivity, suggesting that photoreceptor cell death could occur independently of RPE cell death in response to oxidative stress in this model (Figures 5d–f). Collectively, our data show that Nec-1 inhibits RPE cell death *in vivo,* which indicates that RPE cells die predominantly from necroptosis in response to NaIO_3_.

## Discussion

The oxidizing agent NaIO_3_ has been known to induce selective toxicity in RPE cells. Here, we show that RPE cells die mainly from necroptosis in response to NaIO_3_ *in vitro* and *in vivo*. We found that NaIO_3_ induces hallmarks of necroptosis in RPE cells *in vitro*, such as RIPK3 aggregation and HMGB1 nuclear release. RIPK1 and RIPK3 inhibitors, but not pan-caspase or pyroptosis inhibitors, prevent NaIO_3_-induced RPE cell death. RPE cell necroptosis was confirmed *in vivo* by TUNEL assay, active caspase-3, and HMGB1 staining in NaIO_3_-induced RPE degeneration model. RIPK3 aggregation in response to NaIO_3_ was observed in RPE using novel RIPK3-GFP mouse lines. Furthermore, Nec-1 inhibits RPE cell death in response to NaIO_3_ *in vivo*. Our results indicate that RPE cells die mainly from necroptosis in response to oxidizing agents, which may have implications in the mechanism and therapeutics of dry AMD, especially GA.

### RPE necroptosis in RPE cells in response to sodium iodate *in vitro*

Owing to the critical role of oxidative stress in AMD, numerous efforts have been focused on studying the mechanism of oxidative stress-induced RPE cell death as an approach to decipher the mechanism of AMD pathogenesis. Using either an H_2_O_2_ or tBHP model, researchers attributed oxidative stress-induced RPE cell death mostly to apoptosis. Our published data have shown that H_2_O_2_ and tBHP, induce oxidative stress and RPE necroptosis *in vitro.*^17^ Here, we extended our study to analyze RPE cell death in response to NaIO_3_, a chemical that induces reactive oxygen species production and is selectively toxic to RPE cells.^23,27^ We observed hallmarks of necroptosis in RPE cells in response to NaIO_3_ *in vitro*, including PI membrane permeability, RIPK3 activation, and HMGB1 release from the nucleus. These results are consistent with our results from H_2_O_2_ and tBHP treatments. Furthermore, we discovered that 200 *μ*M Nec-1 or Nec-5, which inhibit RIPK1 directly or indirectly, respectively, protect NaIO_3_-induced RPE cell death. This observation is consistent with a previous report by Balmer *et al*.^18^ However, Nec-7, which targets other necrotic pathways independent of RIPK1, fails to rescue the cells. At the molecular level, activation of RIPK1 and RIPK3 signaling is a hallmark of necrosis.^28–30^ Upon induction of necrosis, RIPK3 forms a complex with RIPK1 that results in necrosome formation. NaIO_3_ induced distinct points of RIPK3 aggregation localized in cell periphery. Use of an RIPK3-specific inhibitor GSK’872 further confirmed the involvement of RIPK3 in NaIO_3_-mediated necroptosis. These observations suggest that NaIO_3_-mediated RPE cell death is dependent on RIPK1 and RIPK3.

RIPK3 aggregation and activation have been recently shown to activate the inflammasome under certain conditions,^20,31^ and inflammasome activation has been implicated in the AMD pathogenesis.^32–35^ Inflammasome activation could lead to the induction of pyroptosis, a type of cell death that depends on caspase-1 activation. Canonical inflammasome activation was not detected by ASC-GFP visualization, while a caspase-1-specific inhibitor Ac-YVAD failed to rescue NaIO_3_-induced RPE cell death, arguing against pyroptosis under these conditions. On the other hand, pan-caspase inhibitor z-VAD experiments argue against significant apoptosis under NaIO_3_ treatment. Taken together, our data support the induction of RIPK1/RIPK3-dependent necroptosis in RPE cells under oxidative stress.

### Mechanism of RPE cell death response to sodium iodate and oxidative stress *in vivo*

There have been numerous histological studies on the *in vivo* effect of NaIO_3_ in different animal models.^26,36–39^ Damage to the retina after NaIO_3_ administration has been shown to be dose-, route-, and time-dependent. Morphological examination of the retina after 100 mg/kg NaIO_3_ administration has indicated RPE depigmentation, swelling, and vacuolization, suggesting RPE necrosis in this model.^23,40^ Evaluation of different doses of NaIO_3_ (15–70 mg/kg) has shown a progressive increase in RPE and photoreceptor damage with increasing dosage, and the dose below 10 mg/kg has been shown to have little effect on the retina.^24,41–43^ The common histopathological changes associated with NaIO_3_ administration include: discontinuity of the RPE layer, disrupted structure of the outer and inner photoreceptor segments, and macrophage infiltration. Destruction of the blood–retina barrier visualized as degradation of ZO-1 protein has been previously described as a result of NaIO_3_ administrations in rabbits.^44^ It was also reported that the RPE damage is most pronounced in the central region of the retina, with relatively little degeneration in the peripheral retina.^43^ However, detailed molecular studies on how RPE cells die in this model have not been performed. We adopted a retro-orbital injection of low-dose NaIO_3_ (20 mg/kg) to study RPE cell death in mice, with the intent to capture the early molecular events leading to RPE death.^24–26^

Our morphological analyses showed that RPE cells start to lose pigmentation at 24 h, and appear swollen, vacuolated, and start breaking off from the RPE layer at 48–72 h post NaIO_3_ administration. More severe RPE damage was observed in the central region of the retina compared with the peripheral regions. We also used *in vivo* PI staining, TUNEL assay, and active caspase-3 staining to distinguish between necrosis and apoptosis in RPE and photoreceptor cells. We found that mouse RPE cells become PI-positive and TUNEL-positive but cleaved caspase-3-negative, while photoreceptors became TUNEL- and cleaved caspase-3-positive. These observations suggest RPE necroptosis and photoreceptor apoptosis in response to NaIO_3_ *in vivo*. RPE necroptosis in this model was further confirmed by HMGB1 release from the nucleus and RIPK3 aggregation using our pVMD2-RIPK3-GFP indicator mice. Moreover, Nec-1 was able to rescue RPE but not photoreceptor cell death *in vivo* in this model. Taken together, our data provide compelling evidence that RPE cells die mainly from necroptosis in response to NaIO_3_ *in vivo*.

Our data that RPE cells die from necroptosis *in vitro* and *in vivo* is consistent with histopathological analyses in several other AMD animal models and human AMD samples. The SOD family proteins constitute a major component of the antioxidant system. Senescent *Sod1*^−/−^ mice displayed RPE vacuolization, one of the common morphological hallmarks of necrotic cells.^45^ Similarly, RPE hypopigmentation, vacuolization, and atrophy were observed in a ribozyme AAV virus-generated *SOD2* knockdown mouse model.^46^ NRF2 regulates expression of many antioxidant/detoxification genes.^47^ *Nrf2*^*−/−*^ mice develop age-dependent RPE degeneration, spontaneous CNV, and subretinal inflammatory protein deposits.^48^ The RPE cells were highly vacuolated with membranous debris. Carboxyethylpyrrole is a unique oxidation fragment of docosahexaenoic acid found in AMD drusen and in plasma samples from AMD patients.^49,50^ Carboxyethylpyrrole-MSA-immunized mice were recently established as a model for studying geographic RPE atrophy.^51^ Features of RPE necrosis, including vacuolization, swelling, cell lysis, and nuclear pyknosis were observed.

Clinicopathological studies have defined the RPE alterations in GA.^52–54^ RPE rounding, sloughing, and entrapment of RPE-derived fragments within basal deposits have been observed in GA samples, supporting necrosis as a mechanism for RPE cell death in GA. Future studies should focus on confirming the molecular nature of RPE cell death in AMD animal models and human AMD samples. RPE necroptosis may have significant implications in AMD. As evidenced in this study, necroptosis inhibitors, such as Nec-1, could potentially be used to prevent RPE death in AMD and GA.

## Materials and Methods

### Generation of RIPK3-GFP transgenic mouse lines

Human RIPK3 cDNA was amplified from the pcDNA3.1 plasmid encoding HA-3xFlag-RIPK3-GFP using the following primers: 5′- AAAAAAGTCGACATGGATTATAAAGATGATGATG-3′ and 5′- AAAAAAGTCGACGAGCTCTAGCATTTAGGTGCA-3′. After digestion with *Sal*I, the purified PCR product was cloned into pVMD2-rtTA vector to replace rtTA.^55^ Linearized vector was used for transgenic injection. Three lines with germline transmission were obtained. These mice are viable, with RPE-specific transgene expression confirmed by western blot and GFP staining. Western blot was performed as described elsewhere.^56^ The antibodies used were: primary anti-GFP (1 : 1000, Aves, Trigard, OR, USA) and secondary IRDye 680RD donkey anti-chicken (1 : 5000, LiCor, Lincoln, NE, USA).

### Sodium iodate injections

For retro-orbtial NaIO_3_ injection, six -week-old C57BL/6J mice of both sexes or pVMD2-RIP3-GFP mice were anesthetized with ketamine/xylazine cocktail; in addition, a topical analgesic was used with 0.5% proparacaine solution in PBS. Sterile freshly prepared 1% NaIO_3_ solution was used for injection via the retro-orbital sinus at 20 mg/kg body weight.^57^ Control mice were injected with PBS.

### Histology and immunohistochemistry

Ocular tissue was fixed in weak formalin Davidson’s solution, embedded in paraffin, and sectioned at 5 *μ*m. Paraffin was removed with orange terpenes (Histoclear, National Diagnostics, Atlanta, GA, USA) and tissue rehydrated through graded ethanol prior to downstream applications. For conventional pathology, tissue sections were stained with Weigert’s Iron Hematoxylin and Eosin Y. For immunostaining, ocular tissue was subjected to heat-mediated antigen retrieval using epitope recovery buffer (Electron Microscopy Sciences, Hatfield, PA, USA), washed with PBS, and permeablized with 0.2% Triton X-100 in PBS. After blocking in 10% normal goat serum, primary antibody was applied and samples were incubated overnight (anti-HMGB1, 1 : 100, Cell Signaling, Danvers, MA, USA; anti-cleaved caspase-3, 1 : 200, Cell Signaling) or for 48 h (Anti-GFP, 1 : 500, Aves) at 4 °C. Alexa Fluor 594-conjugated rabbit anti-mouse (1 : 800) or Alexa Fluor 488 goat anti-chicken (1 : 800) secondary antibodies (Life Technologies, Carlsbad, CA, USA) was incubated with tissue sections for 1 h at room temperature. After washing with PBS, tissue sections were mounted with DAPI containing mounting medium (Vector, Burlingame, CA, USA) and analyzed under fluorescence microscope.

### Semi-thin section preparation and methylene blue staining

Enucleated mouse eye globes were fixed in weak formalin Davidson’s Solution for 4 h at room temperature, followed by soaking in 70% ethanol for 1 h. After removing tissue from ethanol, the eye was gross cut in the sagittal plane (removing ~20% of the globe) with the lens not removed. The tissue was further dehydrated through 95% and absolute ethanol. Next, the tissue was soaked in acrylic resin (Unicryl, BB International, Cardiff, UK) for 1 h at room temperature with shaking. After 1 h, the resin was removed, fresh resin was added, and the tissue was allowed to shake overnight at room temperature. The next day, the tissue was transferred to an embedding mold and fresh acrylic resin was added. The tissue was baked in the embedding mold for 48 h at 60 °C. After baking, 1-*μ*m sections were cut with a glass knife on an ultra-microtome (Reichert Ultracut S, Leica Microsystems, Wetzlar, Germany). Sections were floated on drops of water on a microscope slide and baked on a slide warmer (~55 °C) until water evaporated. For staining, 0.2% methylene blue diluted in 95% ethanol was added to the sections while the slides were still on the slide warmer. After 15–30 s, slides were rinsed with deionized water, flooded with orange terpene (Histoclear, National Diagnostics), tapped dry on a paper towel, and mounted with permanent mounting medium (Permount, Fisher Chemical, Waltham, MA, USA).

### TUNEL assay

Terminal deoxynucleotidyl transferase dUTP nick end labeling (TUNEL) was used to analyze cell death *in vivo*.^58^ Briefly, paraffin was removed from the ocular sections by orange terpene (Histoclear, National Diagnostics) and rehydrated through a graded ethanol series to double distilled water. Tissue sections were then permeabilized in freshly prepared 0.1% Triton X-100, 0.1% sodium citrate solution. Terminal deoxynucleotidyl transferase solution was prepared according to manufacturer’s directions (Roche Applied Science, Mannheim, Germany) and incubated with the tissue sections for 60 min at 37 °C. Samples were then washed with PBS, mounted with DAPI containing mounting solution (Vector), and analyzed under the fluorescent microscope (Nikon, Tokyo, Japan).

### Sclera-RPE flatmount ZO-1 and PI staining

For flatmount ZO-1 staining, enucleated mouse eye globes were washed in PBS. The anterior chamber, lens, and neuroretina were removed and the eyecup was fixed in 100% methanol for 30 min at room temperature. Next, the eyecup was incubated in blocking buffer containing 0.5% Triton and 5% horse serum (Gibco, Waltham, MA, USA) in PBS for 1 h at room temperature. Primary antibody against zonula occludens (ZO)-1 (1 : 100, Life Technologies) was incubated with the eyecup at 4 °C overnight. Alexa Fluor 594-conjugated goat anti-rabbit (1 : 800) was prepared in PBS and incubated with cells for 1 h at room temperature. After wash with 1× PBS, the eyecup was flat-mounted with DAPI containing mounting medium for fluorescence (Vector) and analyzed under a fluorescence microscope (Nikon). Flatmount PI staining was performed similarly as described elsewhere.^59^ PI (0.5 *μ*g, Life Technologies) was injected through retro-orbital injection at 15 min before killing the mice. The eye globes were enucleated, anterior part was removed, and retinas were flat-mounted for fluorescence microscopy.

### Cell culture, transfection, treatments, and MTT assay

Human RPE cells (ARPE-19, CLR-2302, ATCC, Manassas, VA, USA) were cultured in DME/F-12 medium (HyClone, Logan, UT, USA) supplemented with 10% FBS (HyClone) and 1× Penicillin-Streptomycin solution (HyClone) at 37 °C in 5% CO_2_. Cells were treated with: 50 *μ*M z-VAD (Sigma-Aldrich, St Louis, MO, USA), 200 *μ*M necrostatin-1, -5, -7 (Enzo Life Sciences, Farmingdale, NY, USA), 50 *μ*M caspase-1 inhibitor Ac-YVAD (Sigma-Aldrich), or 3 *μ*M GSK’872 (Millipore, Billerica, MA, USA). Cells were treated with 10 mM of sodium iodate (Sigma-Aldrich) in PBS (Gibco).

Cell transfection was performed using Lipofectamine LTX (Life Technologies, Farmingdale, NY, USA). Briefly, 1 *μ*g of HMGB1-YFP, ANT1-RFP, RIPK3-GFP, or PYCARD-GFP plasmid DNA was mixed with 5 *μ*l of Lipofectamine LTX. The complex was added to the ARPE-19 cell cultured in 4-chamber glass slide 20 min later. Expression of the recombinant proteins was visualized upon NaIO3 treatment after 24 h under a fluorescent microscope.

To assess cell viability, MTT assay was performed as described previously.^60^ In short, ARPE-19 cells were incubated with 1 mg/ml of MTT reagent (Sigma-Aldrich) for 2 h in standard cell culture conditions. Developed MTT crystals were dissolved in DMSO (Sigma-Aldrich) and analyzed at the 96-well plate reader by measuring absorbance at 540 nm.

### Mouse vitreous humor collection and ELISA analysis

Vitreous humor collection was performed as described previously.^61^ In brief, a linear incision was made in the cornea and the anterior chamber fluid was removed. Next, pressure was applied at the external surface of the sclera, and the lens was pushed forward through the corneal incision. The vitreous gel was placed in the centrifugation tube and dissolved in PBS (Gibco). Level of HMGB1 in mouse vitreous humor was measured by enzyme-linked immunosorbent assay (ELISA) kit according to the manufacturer’s manual (Elabscience, WuHan, China). Briefly, the samples were added to the plate pre-coated with anti-HMGB1 antibody and incubated for 90 min at 37 °C. After removing the samples, biotinylated detection antibody was added. After incubation for 1 h at 37 °C and PBS wash, HRP conjugate was added and the samples were incubated for 30 min at 37 °C. After washing with PBS, substrate reagent was added to the plate for 15 min at 37 °C, and the reaction was stopped by the stop solution. The plate was analyzed on a micro-plate reader at 450 nm (Molecurar Devices, Sunnyvale, CA, USA).

### Statistics

Each experiment was repeated at least three times. Student’s *t*-tests were used to determine statistical significance between groups. *P*-values of less than 0.05 were considered to be statistically significant.

## Abbreviations

AMD: age-related macular degeneration
ANT: adenine nucleotide translocator
ASC: apoptosis-associated speck-like protein containing a carboxy-terminal CARD
ATP: adenosine triphosphate
ELISA: enzyme-linked immunosorbent assay
GA: geographic atrophy
GFP: green fluorescent protein
H_2_O_2_: hydrogen peroxide
HMGB1: high mobility group box 1
NRF2: nuclear factor (erythroid-derived 2)-like 2
PI: propidium iodide
RFP: red fluorescent protein
RIPK: receptor interacting protein kinase
RPE: retinal pigment epithelium
SOD1: superoxide dismutase 1
tBHP: tert-butyl hydroperoxide
TUNEL: terminal deoxynucleotidyl transferase dUTP nick end labeling
YFP: yellow fluorescent protein
ZO-1: Zona occludens 1.
