# Supplementary figures and images for: Retinal pigment epithelial cell necroptosis in response to sodium iodate
Source: Cell Death Discov. 2016 Jul 4;2:16054–. doi: 10.1038/cddiscovery.2016.54 (PMC4979458; doi:10.1038/cddiscovery.2016.54)

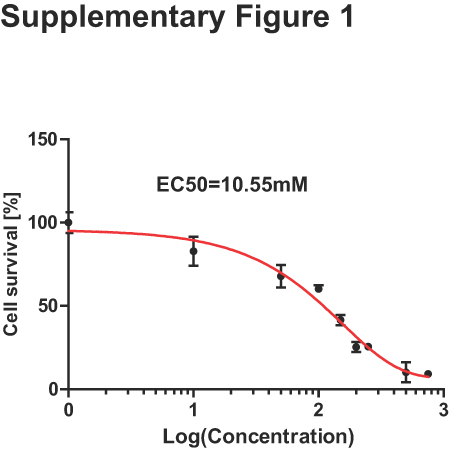

Supplement: Supplementary Figure 1 [file cddiscovery201654-s1.jpg]
