# Supplementary material for: Retinal pigment epithelial cell necroptosis in response to sodium iodate
Source: Cell Death Discov. 2016 Jul 4;2:16054–. doi: 10.1038/cddiscovery.2016.54 (PMC4979458; doi:10.1038/cddiscovery.2016.54)
Supplement: Supplementary Information [file cddiscovery201654-s2.doc]

Viability of ARPE-19 cells was determined 24 hours after inducing cell death with different concentrations of sodium iodate. Data represents the mean (S.E.M.) of 3-5 independent experiments.
